# Loss of Rituals, Boundaries, and Relationship: Patient Experiences of Transition to Telepsychotherapy Following the Onset of COVID-19 Pandemic

**DOI:** 10.3389/fpsyg.2022.835214

**Published:** 2022-03-24

**Authors:** Andrzej Werbart, Linda Byléhn, Tuva Maja Jansson, Björn Philips

**Affiliations:** Department of Psychology, Stockholm University, Stockholm, Sweden

**Keywords:** remote psychotherapy, online therapy, communication technology, patient experiences, personality orientation, therapeutic boundaries, therapeutic relationship, thematic analysis

## Abstract

Telepsychotherapy is an increasingly common way of conducting psychotherapy. Previous research has shown that patients usually have positive experiences of online therapy, however, with large individual differences. The aim of this study was to explore patients’ experiences of transition from in-person psychotherapy sessions to telepsychotherapy during the COVID-19 pandemic, as well as variation in the experiences with regard to the patients’ personality orientation. Seven psychotherapy patients in Sweden were interviewed and the transcripts were analyzed using thematic analysis. Additionally, the participants were asked to rate their dissatisfaction/satisfaction with the transition, how hindering/helpful the transition was, and how unsafe/safe they felt after the transition in comparison to before. Personality orientation on relatedness or self-definition was assessed applying a self-assessment instrument (Prototype Matching of Anaclitic-Introjective Personality Configuration; PMAI). The participants experienced telepsychotherapy as qualitatively different from in-person psychotherapy. They reported several essential losses: the rituals surrounding therapy sessions were lost, including the transitional time and space between their every-day life and the therapy sessions, less therapeutic work was done, the therapists could lose their therapeutic stance, the sense of rapport was impaired, and the patients felt less open and emotionally available. On the other hand, some patients could feel freer online. As six of the participants had an anaclitic personality orientation, the present study could especially contribute to the understanding of how patients with strong affiliative needs and fear of abandonment experience the transition to meeting their therapists *via* communication technology. The participants’ self-ratings showed that they were only marginally dissatisfied with the transition and experienced the transition as slightly hindering, whereas they felt rather safe after the transition, indicating low concordance between qualitative and quantitative evaluations. New studies are needed to explore the introjective patients’ experiences of the transition. An essential topic is also to collect evidence and to test how the impaired sense of rapport when using communication technology can be remedied by adequate, patient-tailored interventions, a topic that has to be included in psychotherapy education and training.

## Introduction

In spring 2020 the world was hit with the COVID-19 pandemic, which took many lives and forced the whole world to readjust to a new reality. With the aspiration to minimize spread of infection, restrictions were made worldwide. In March 2020, the Public Health Agency of Sweden (Folkhälsomyndigheten [The Public Health Agency of Sweden], 2020) recommended that everyone that could should work from home, as well as urging workplaces to find remote alternatives to in-person interactions. Simultaneously, the World Health Organization (WHO, 2020) declared the psychological consequences of the pandemic as a public health issue, stressing that it was critical that people in need of mental health treatment would still have access to treatment in an infection-proof setting, and the United Nations (2020) recommended mental health services to be delivered online. Accordingly, the COVID-19 pandemic has brought with it a rapidly increased use of telepsychotherapy, confronting many patients and therapists with the need of reliance, for the first time, on means of communication technology.

American Psychological Association (2013) defined telepsychology as the provision of psychological services using telecommunication technologies. According to a systematic review (Yue et al., 2020), remote care services play a central role in treating mental health consequences of infectious disease outbreaks, such as COVID-19 pandemic; however, there is a need of recognizing the limitation of such teleservices. Previous research shows that different modalities of remote psychotherapy (using phone, audio or video internet connection, or chat) are effective in reducing symptoms in a wide range of mental disorders, such as depression, anxiety, PTSD, and panic disorder (Foroushani et al., 2011; Andersson et al., 2019a,b; Bennett et al., 2020; Lindegaard et al., 2020; Lindqvist et al., 2020). At a group level, patients’ experience of telepsychotherapy is often positive; nevertheless, there is a large variation at the individual level (Simpson, 2001; Simpson et al., 2005; Leibert et al., 2006; Bennett et al., 2020; Watts et al., 2020).

After the outbreak of COVID-19 pandemic, a growing number of studies focused on effects of forced transition to telepsychotherapy. In a survey on the experiences of 141 therapists in United States who transitioned to providing video therapy during the pandemic (Aafjes-van Doorn et al., 2021) the responders reported some anxiety and self-doubt. However, most felt that online sessions had a sufficient working alliance and a strong real relationship. Therapists with more online therapy experience, lower levels of self-doubt and anxiety, and those who experienced a strong online real relationship during the pandemic, or thought their patients viewed it positively, tended to be more accepting of video therapy. Likewise, a survey among 150 Israeli therapists (Nuttman-Shwartz and Shaul, 2021) showed that the more experience therapists had, the less they perceived the current situation as a threat to both themselves and their patients (“shared traumatic reality”). According to a worldwide survey among 1,490 psychodynamic therapists (Gordon et al., 2021), the therapist’s empathy, warmth, wisdom, and skillfulness, and the patient’s motivation, insightfulness, and level of functioning were considered as more important to effective psychotherapy than the differences between in-person and remote therapy.

In contrast to these survey results, therapists report several hindering, both practical and emotional, factors in the transition to telepsychotherapy. Safeguarding the therapeutic frame became more problematic. It could be difficult for the patients to have access to an undisturbed room with a stable internet connection. They could be distracted by things in their everyday life or be engaged in doing household chores during the remote session, turning therapy into a practical ingredient in everyday life, where the emotional closeness was lost (Dolev-Amit et al., 2021; Rizq, 2020). There was a risk of developing a certain relational distance when the communication technology provided limited sensory information about the patients’ emotional presence (Ronen-Setter and Cohen, 2020). This might in turn also make the therapist more distant (Dolev-Amit et al., 2021).

Furthermore, the therapists and their patients might have discordant views of the transition. For example, according to an Austrian survey (Probst et al., 2021) the therapists reported using fewer therapeutic interventions in remote therapy than in in-person therapy, whereas the patients did not report any differences. Additionally, patients with different characteristics might have different experiences of the forced transition to telepsychotherapy. Some of them can experience telepsychotherapy as impersonal and distanced, whereas others can be less self-conscious and more open in remote sessions (Simpson, 2001; Simpson et al., 2005; Chen et al., 2021). Patients with generalized anxiety disorder in cognitive behavioral therapy *via* videoconferencing reported a better working alliance than patients in face-to-face psychotherapy (Watts et al., 2020). For some patients with severe depression or PTSD, the physical distance in telepsychotherapy can contribute to them feeling safer and more confident in the therapist, whereas patients with greater needs for the therapists’ presence for emotional regulation and safety might feel more challenged in telepsychotherapy (Chen et al., 2021).

Extensive research has shown that personality characteristics influence what patients are looking for when seeking psychotherapeutic help, how they make use of therapy, and how helpful different forms of therapy are for them (Blatt et al., 2001; Blatt and Shahar, 2004a,b; Blatt and Luyten, 2009; Levander and Werbart, 2012; Werbart and Levander, 2016; Werbart et al., 2020). According to Blatt’s (2008) empirically supported theoretical “double helix” model, psychological development is a lifelong interplay between two basic dimensions in human experiences: the anaclitic orientation on interpersonal relatedness (ability to develop empathic, reciprocally attuned relationships) and the introjective orientation on self-definition (ability to establish a coherent, realistic, differentiated and positive sense of self). A good balance between these two dimensions is a prerequisite for mental well-being; in contrast, different forms of psychopathology reflect an exaggerated and distorted preoccupation with one of them (Blatt and Luyten, 2009; Luyten and Blatt, 2013; Luyten et al., 2013). Higher levels of anaclitic and introjective orientation roughly correspond to attachment anxiety and attachment avoidance, respectively (Meyer and Pilkonis, 2005; Mikulincer and Shaver, 2007; Luyten and Blatt, 2011), whereas low levels on both dimensions, together with an anaclitic-introjective balance, are connected with secure attachment (Werbart et al., 2016).

Predominantly anaclitic patients often seek psychotherapy for relational problems and usually adapt fast to the psychotherapeutic setting. Their main complaints include a sense of helplessness, loneliness, and a fear of abandonment. In therapy, they look for warmth and care, and they are often helped by more supporting interventions. Predominantly introjective patients usually seek therapy for issues regarding control, performance, and not being in contact with their emotions. Their main complaints center on excessive self-demands, feelings of inferiority, and fear of failure and criticism. They tend to keep others at a distance and make the best use of interpretative interventions in psychotherapy. Consequently, anaclitic patients seem to value and are more responsive to the quality of the therapeutic relationship, whereas introjective patients lay emphasis on increasing their understanding of themselves (Blatt and Shahar, 2004b; Blatt and Luyten, 2009; Levander and Werbart, 2012; Luyten and Blatt, 2013; Werbart and Levander, 2016; Werbart et al., 2017, 2020; Hennissen et al., 2020). Anaclitic patients seem to benefit more from therapy with a greater relational focus on interaction in a face-to-face setting, where the therapist and patient can see one another, whereas introjective patients seem to benefit more from psychoanalytic therapy with a greater focus on insight and self-reflection, lying on the couch, where the patient does not see their therapist (Blatt et al., 1988, 2007, 2010; Blatt, 1992; Blatt and Ford, 1994; Blatt and Shahar, 2004b). Furthermore, anaclitic patients are described as profiting from a warm and caring therapeutic relationship, whereas introjective patients are described as striving to interpret the therapist’s non-verbal expressions and adjust to them in order to maintain their sense of control, ultimately avoiding shame and guilt (Blatt, 1991, 2008; Blatt and Ford, 1994; Blatt and Shahar, 2004a,b; Blatt et al., 2010). Thus, personality orientation can affect how well patients function in different forms of psychotherapy.

Experiences from clinical practice and supervision, as well as recent publications at the onset and during the COVID-19 pandemic (cf. Chen et al., 2021; Ehrlich, 2021; Essig and Isaacs Russell, 2021; Isaacs Russell, 2021) indicate that different patients reacted different to the transition. Some of them were lacking the direct in-session contact, physical presence at the same place, and the own time on the way to and from the therapist’s office, and found it difficult to maintain a good enough therapeutic relationship online. Others were relieved not to have to travel, not to sit in the same room, and they could “open up” more than previously in the ordinary psychotherapy setting.

To sum up, the patient perspective on the transition to telepsychotherapy is still underexplored and most of the recent publications are based on therapist reports (e.g., Chen et al., 2021; Rizq, 2020; Essig and Isaacs Russell, 2021; Isaacs Russell, 2021; Sayers, 2021; Weinstein, 2021). There is still an urgent need of more research on which patient characteristics can contribute to more positive or more negative experiences of the transition, how these characteristics affect the treatment process and outcome, as well as in what circumstances telepsychotherapy might be a viable alternative to in-person psychotherapy. COVID-19 pandemic gave us a unique opportunity to explore for which patients the customary in-person psychotherapy setting with the patient and the therapist co-present in the same room is the treatment of choice, and for which patients remote therapy using communication technology might be more favorable.

The present study aims to examine patients’ experiences of the transition from conventional in-person psychotherapy to telepsychotherapy, using video or audio communication technology, during the COVID-19 pandemic, and how the patients’ personality orientation might influence these experiences. Based on previous research we assume that the effects of transition from in-person psychotherapy setting to telepsychotherapy will be experienced as more positive and facilitating by the predominantly introjective patients with their main focus on autonomy and performance, and as more negative and hindering by the predominantly anaclitic patients with their main focus on relatedness and intimacy.

## Materials and Methods

### Participants

The initial goal was to recruit 12–15 participants for this study, with an equal amount of persons with anaclitic and introjective personality orientation, respectively. However, due to difficulties in finding enough participants who met all the inclusion criteria during the time frame of the study, the number of included participants stayed at seven. Inclusion criteria were that the participants must have been in psychotherapy during the COVID-19 pandemic 2020–2021 with a licensed psychologist or psychotherapist at least once a week during at least 4 weeks before transitioning to at least four sessions of telepsychotherapy. Recruitment was carried out through posters and advertising in social media, with a Facebook advert that reached 25,000 users. Despite the large number of people who viewed the advert, only 15 persons signed up for the study and eight of these were excluded because they did not fulfill all inclusion criteria. Of the seven included participants, five were female and two were male. Participants’ age ranged from 27 to 51 (*M* = 33). Three participants were in cognitive-behavioral therapy, further three in psychodynamic therapy, and one participant did not know the therapeutic orientation. In five cases the transition to telepsychotherapy was initiated by the therapist and in two cases by the patient. Four participants were still in telepsychotherapy at the time of the interview, and one was back to in-person psychotherapy. Three participants were no longer in psychotherapy. All participants had been in telepsychotherapy through video link. Two of them had only video sessions, whereas five participants had both video and telephone sessions, of which two used both communication channels equally, two mostly used video and in exceptional cases telephone, and one participant initially used video link and later on preferred telephone. No data on the focus in psychotherapy were collected. All participants gave their informed consent before inclusion in the study. The consent forms were collected online applying the platform Survey and Report, provided by the Stockholm University for research with human subjects.

### Data Collection

Data were collected in March–April 2021 through semi-structured interviews, self-report scales regarding the experience of the transition to telepsychotherapy, and for assessment of personality organization. The interviews were conducted online using Zoom platform audio, lasted about 45 min, and were audio-recorded. The interviewers were the second and the third author, at the time of the study students in the final semester of the Swedish 5-year clinical psychology program (psychodynamic orientation). Each interviewer conducted three or four interviews. The self-report instruments were e-mailed to the participants, who completed them and subsequently brought them to the interview. When the qualitative interview was completed the participant reported their self-ratings.

### Interviews

An interview protocol was developed to address the participants’ experiences of the transition. The aim of the semi-structured interviews was to collect the participants’ accounts covering the following areas:

• What kind of communication technology was used?
• How the decision on the transition to remote therapy was made.
• Positive and negative experiences of the transition.
• Hindering and helpful aspects of the transition.
• How the transition affected the therapeutic relationship, the therapy process and the experienced outcome.

The participants were asked to give specific examples and to elaborate their answers. The interviews ended with a question if there are another experienced aspects of the transition that the participant and the interviewer did not talk about.

### Self-Ratings of Experiences of the Transition to Telepsychotherapy

In order to have some quantitative self-assessment of the participants’ experiences of the transition they were asked to assess on 7-point Likert scales (a) how dissatisfied/satisfied they were with the transition, (b) how hindering/helpful the transition was, and (c) how unsafe/safe they felt after the transition in comparison to before. These scales were constructed for the present study and were used only for descriptive purposes and not for statistical analyses.

### Prototype Matching of Anaclitic-Introjective Personality Configuration

The participants’ personality orientation was assessed using Prototype Matching of Anaclitic-Introjective Personality Configuration (PMAI), a self-assessment form that presents prototypes for anaclitic and introjective personality orientation (Werbart and Forsström, 2014; Werbart and Levander, 2016). The prototype-matching method generates both categorical and dimensional assessments. The participants were asked to rate on a 5-point Likert scale how well they recognized themselves in each prototype and to specify which of the two prototypes that best corresponded with their own view of themselves. Because our aim was to compare anaclitic and introjective participants, the PMAI results were used to categorize the participants into predominantly anaclitic or predominantly introjective orientation. Cases were sorted as anaclitic or introjective, following the highest score on one of the two dimensions, and following the categorical self-assessment in cases when both dimensions were rated equally.

### Analysis

The data were analyzed applying a rigorous qualitative methodology following Braun and Clarke (2006, 2013) six steps for thematic analysis. This was supplemented with a descriptive analysis of quantitative self-assessments of experiences of transition and of personality orientation.

The first step in the thematic analysis was familiarizing oneself with the data, which included transcribing and reading the material as well as taking notes. The next step was initial coding of the interview transcripts, conducted by the person who did not conduct the coded interview, thus being blind of the participants’ self-assessments and keeping an inductive stance in the analysis. The third step was made jointly by both interviewers, bringing the codes from the individual coding together and developing preliminary themes. These preliminary themes were reworked and discussed to form more comprehensive themes. In the fourth step the themes were evaluated and compared with the initial codes and original data. Main themes and subthemes were sorted and a visual thematic map was created. In the fifth step all themes were defined and labeled with the aim of capturing the essence of the themes while still being clearly differentiated from other themes. The themes were then re-evaluated, comparing them once again with the original data. The sixth step was compiling a preliminary report and selecting quotes best illuminating each theme.

The guidelines from Hill et al. (2005) were used for indicating the frequency of each theme. Themes occurring amongst all or all but one participant were labeled *general*; themes occurring amongst more than half of participants were labeled as *typical*; and themes occurring amongst at least two up to half of the participants were labeled as *variant*.

The thematic analysis was complemented by a categorization of each participant’s overall view of the transition to telepsychotherapy as *positive*, *mixed, and negative*. This judgment was made by the interviewers separately and then discussed until a consensus could be reached.

## Results

All participants described both positive and negative experiences of the transition. However, the participants expressed a general dissatisfaction with the transition, experiencing telepsychotherapy as less effective than regular psychotherapy. All participants uttered a wish to meet their therapist in person, although one participant would have preferred to also have some of the sessions *via* telephone. The qualitative analysis showed that the already established safe relationship with the therapist worked as a buffer during the transition.

### Themes of Transition

Thematic analysis resulted in seven main themes and 14 subthemes, together illuminating different facets of the transition (Table 1). All themes are presented below in order of their frequencies and illustrated by verbatim quotations from the interviews. Each quotation is followed by an indication in square brackets of the participant’ personality orientation ([A] = anaclitic, [I] = introjective). All of the main themes were categorized as general, with an exception for the typical theme *5. Feeling freer*.

**TABLE 1:** Themes and subthemes in the participants’ experiences of transition to telepsychotherapy.

Theme	Frequency			Label
	A (*n* = 6)	I (*n* = 1)	Total (*n* = 7)	
1. Loss of therapeutic rituals	6	1	7	General
1.1. The therapy lost some value	6	1	7	General
1.2. Loss of the therapeutic space	6	1	7	General
2. Less therapeutic work	6	1	7	General
2.1. The therapist lost their therapeutic stance	6	0	6	General
2.2. Being less in focus	3	1	4	Typical
2.3. Blurred therapeutic boundaries and methods	3	0	3	Variant
3. Impaired sense of rapport	6	1	7	General
3.1. Impaired communication	4	1	5	Typical
3.2. Increased relational distance	6	1	7	General
4. Being less emotionally available and open	6	1	7	General
4.1. Feeling less emotionally present	5	1	6	General
4.2. Being less open	4	1	5	Typical
5. Feeling freer	4	0	4	Typical
5.1. The therapy became less demanding	4	0	4	Typical
5.2. Feeling less self-conscious	4	0	4	Typical
6. The online setting was both helpful and hindering	6	1	7	General
6.1. It was more convenient	6	1	7	General
6.2. The technology was hindering	5	1	6	General
7. The therapy became essentially different	5	1	6	General

*Frequencies of participants in each theme and subtheme for anaclitic and introjective participants and totally [labeled following Hill et al. (2005): General = 6–7; Typical = 4–5]; Variant = 2–3.*

#### 1. Loss of Therapeutic Rituals

This general theme captures how the therapy room as a physical and geographical place enhanced the experienced value of the therapy. The participants described how the therapy room became something more than simply a room and that the travel to and from therapy gave opportunity to thoughtfulness and reflection. They reported how routines surrounding their customary therapy became rituals helping them in transition to a more receptive state of mind. Loss of these rituals and of the intermediate room and time made the therapy feel less important and less valuable.

##### 1.1. The Therapy Lost Some Value

Generally, the participants experienced less mental and emotional investment in telepsychotherapy. The material conveys a general feeling that the therapy was less charged when on distance. Customary in-person therapy was experienced as something exceeding everyday chores, and there was a sense of solemnity in the participants’ descriptions of co-present work. Telepsychotherapy was less valued and was perceived as a routine and something to check off a list. The sessions felt like any other remote meeting and lost the quality of being something special.

It was like “okay, I have therapy this Thursday, so then I’ll be going there,” so I scheduled it and made sure that I maybe didn’t have too many demanding businesses afterward… but I could squeeze in that call during lunch at work when we had remote therapy. So then it became less… sort of less valuable. [A]

##### 1.2. Loss of the Therapeutic Space

Generally, the participants experienced that the therapy lacked something essential when the usual therapy room was unavailable. The physical space also created a mental space, a neutral area demarcated from everyday life and allowing all kinds of thoughts and feelings. In telepsychotherapy, the setting always could be affected by something else. The process of traveling from everyday life to the therapist’s office also created a mental process of leaving everyday life behind and entering the time and space of therapy. As expressed by one of the participants: “Like, in a [therapy] room there is much more… emotions. And also that it is somewhere else. That it’s not… like a totally blank sheet that you get to go into.” [A]

#### 2. Less Therapeutic Work

Generally, the participants experienced that the frame alternation restricted the range of therapeutic work. This was not experienced as the therapist’s intention but rather as a consequence of the online mode being new to them as well. Some exercises that had been part of customary in-person therapy fell away, the therapist’s role changed, and the therapeutic boundaries became more ambiguous. There was a feeling that the therapy was lacking a movement forward and that some therapeutic aspects were lost.

##### 2.1. The Therapist Lost Their Therapeutic Stance

Generally, the participants described how the therapist’s approach to them changed in a way implying loss of the therapeutic attitude. The sessions were more like speaking with a friend, and the asymmetry constituting the therapeutic relationship was reduced. The conversations were more like socializing and the therapist became more self-disclosing. Such more friendly conversations could be experienced as less demanding and give a feeling of getting closer to the therapist, even if the participants questioned how therapeutically effective this was. The therapists became less exploring and less confronting, more absent-minded, and involved in something else, as doing cleaning or other home activities. This could be expressed as criticism from the participants, but often regarded as a natural consequence of the changed format.

Well, it actually felt like it came from her! Because she started talking more like… or maybe it was me as well, but it felt a little bit like she was more… that the borders were more blurred for her as well. And that she started talking more like… chill talk. And she told me more about her life… I feel like that’s nice as well, and that it might make me be more relaxed, but maybe it was a bit… I don’t know if it was a huge problem, but it was a bit too much of it. [A]

##### 2.2. Being Less in Focus

Typically, the participants felt that they no longer received the therapist’s full attention. The therapeutic work was less centered on them as individuals and their problems; it was harder to highlight their needs and dare to confront the therapist. A feeling of abandonment colored the material, where participants felt uninteresting and replaceable to their therapist. Feeling no longer prioritized, they could be jealous and disappointed:

When we last spoke it felt a bit like he didn’t remember… like… I have quite a messy life right now, but he didn’t quite remember what my life is like. And then I start to think “Is it because he has gotten more patients now or is it because…” well like “because we are at a distance now?” [A]

##### 2.3. Blurred Therapeutic Boundaries and Methods

As a variant, the participants experienced that the therapeutic frames and working methods became vaguer, as if remote therapy lost its focus and direction. There was also more uncertainty regarding duration and frequency of therapy sessions. The telepsychotherapy was experienced as more indistinct and lacking an explicit agreement about how the therapy was supposed to be carried through.

Maybe it also was a bit unclear what the plan… we didn’t really have a plan or so, I was mostly there and sort of talked about my sorrow and anxiety. But since we didn’t have a real plan it was more like that [a more general conversation]. [A]

He also actually eh… cut down on our time! [laughing] and I haven’t dared to talk with him about that yet! [laughing] … because you usually have 45 min for each session. And the last two times he asked after 30 min about the next time and then at 35 min we closed down… That might not have been as easy for him to do with me in the usual room, I don’t know. [A]

#### 3. Impaired Sense of Rapport

Generally, the participants reported impaired bond with the therapist. They felt that something was lost in the relationship and that it was harder to communicate when they could not see or take in the whole person they were speaking with. It felt harder to reach each other and to meet one another in the dialog, and the feeling of sharing the therapeutic work with their therapist faded.

##### 3.1. Impaired Communication

Typically, the participants experienced the communication was more difficult in online setting and they felt restricted to verbal channels. This made it harder both to convey their message and to perceive what the therapist is communicating. They lacked the capacity to make use of body language and felt that emotions were lost in technologically mediated communication.

I think about that he can’t asses my body language, intonation and facial expressions. … That you’re very much in the words. And that is one of the things I have been working on a lot, to be less “in the words” and more in something else… So it becomes very like verbal. And it’s just like one dimension, to me. [A]

In some way it’s like I have to rely more on what he says. Cause I can’t catch … I can see that he reacts in a way that I KNOW is him expressing empathy …But I don’t get the same feeling of it when I see it on the screen as if I’m in the same room. [I]

##### 3.2. Increased Relational Distance

Generally, the participants described that the telepsychotherapy made the therapeutic relationship rather feeble and it was harder to be close each other. At times the therapist was perceived as dehumanized and unreal. The moments of here-and-now meeting were compromised and this could also change the relationship in general.

It is that it’s quite [laugh] dehumanizing. That the screen becomes like a… threshold or a wall. Like it’s not the same contact as when you meet physically. Then it’s more like … yes you could say it’s almost like an avatar. [A]

And then it becomes like that with the therapist as well. That that person becomes a bit unreal. If you’re only a voice, or only a little flat… But I’m very like… I know how someone smells, I know how they move, and then it becomes an entirety …. If I can’t get the whole it gets tough for me. [A]

#### 4. Being Less Emotionally Available and Open

Generally, the participants experienced that it was more difficult to access the emotional content and to share one’s experiences with the therapist. Online sessions became more intellectual and it was easier for the participants to avoid emotionally challenging topics.

##### 4.1. Feeling Less Emotionally Present

Generally, the participants experienced less of emotional presence in the online sessions. They felt less vulnerable and could more easily distract themselves. This resulted in less of emotional content in the therapy and it took more effort to reach one’s own susceptibilities.

I think it’s fairly okay… I mean it’s nice being able to… but it is also a distraction [laugh] in the conversation, me trying to distract myself by doing something else… So I guess it feels good at that moment, but I realize that I’m distracting my thoughts and my body from troublesome feelings. [A]

##### 4.2. Being Less Open

Typically, the participants felt less open in online setting than they had felt previously in customary in-person sessions. It was easier to keep in hiding and not to share some of their emotions and aspects of their personality with the therapist: “It might make me restrain myself more. This counteracts the therapeutic work of getting in touch with the feeling. That I try to actively inhibit it, because it doesn’t feel as natural to express that emotion.” [I]

#### 5. Feeling Freer

Typically, the participants felt less self-conscious and restricted in remote sessions. They became freer in their thoughts and reasoning and they pondered less about the therapist’s reactions. It was easier to talk and associate freely using only audio link and not being able to see their therapist. However, it was mostly the intellectual exchanges that became freer.

##### 5.1. The Therapy Became Less Demanding

Typically, the participants felt freer due to the experience of reduced demands in telepsychotherapy. They did not experience the same pressure to deliver and claim their right to be there. The relationship with the therapist became more relaxed and the conversational climate more forgiving.

Also, I felt less stressed before the meetings, with planning and such… that I didn’t feel like “now I have to get as much as possible out of this since I took my time getting here” and so forth. So in this way it was a bit less demanding somehow when we had remote therapy. [A]

##### 5.2. Feeling Less Self-Conscious

Typically, participants experienced less self-consciousness in remote sessions. Due to increased difficulty when interpreting the therapist’s reactions, the participants felt that they could not adjust to the therapist to the same extent as before. The tools they previously used to check how they were perceived by their therapist were lost, releasing them of being preoccupied with the therapist’s reactions and opinions.

I mean, I could feel that when you’re talking over the phone, I didn’t have a visual impression and that made my thoughts flow more freely in a way. Because otherwise I tend to read into body language and facial expressions, and the other person’s reactions. In a way it was somewhat relieving not having to do that. [A]

#### 6. The Online Setting Was Both Helpful and Hindering

Generally, the participants felt that the technologically mediated communication was both helpful and hindering. The transition influenced first and foremost the therapeutic setting and the practical arrangement, but the very experience of the therapy was also affected.

##### 6.1. It Was More Convenient

The participants generally expressed that the most conspicuous advantage of the remote setting was that the therapy became more accessible and flexible, both in terms of travel time and booking of sessions. The sessions that otherwise would have been canceled could still be fulfilled in the online setting. However, these advantages were generally experienced as not so crucial.

A session nowadays really takes 45 min, but… I mean when I had to get there and so on it would take about one and a half hour or maybe more because I wanted to make sure I was on time. So the biggest thing is… regarding time and effort it’s better. [A]

##### 6.2. The Technology Was Hindering

Typically, the participants described that the use of communication technology could have a negative impact on the therapy. The need of equipment, technical difficulties and disrupted connection could obstruct the therapeutic work and make the therapy less available.

I think it could have gotten worse due to, you know, technical disruptions… and I feel like it might be irritating to him, of course. Or like “oh, now FaceTime doesn’t work, so we have to take it on the phone!”… so I think it had a negative impact. [A]

#### 7. The Overarching Theme: The Therapy Became Essentially Different

Generally, the participants experienced telepsychotherapy as qualitatively different from customary in-person therapy. They described that the same therapeutic interventions generated completely different feelings when performed at a distance. According to one participant: “I do not get the same feeling when the feedback is *via* Zoom somehow. Even though I know that it is well meant, it doesn’t feel as good somehow… [Even if] the verbal content is still exactly the same.” [I]

This experience was difficult to define and put into words for the participants, and did not necessary imply that telepsychotherapy was inferior or superior to the usual setting, only that it was a clear but indefinable and elusive difference: “It is something… Another tone, or another input. And perhaps mostly different dynamics.” [A]

### Interconnections Between the Themes

**FIGURE 1:**
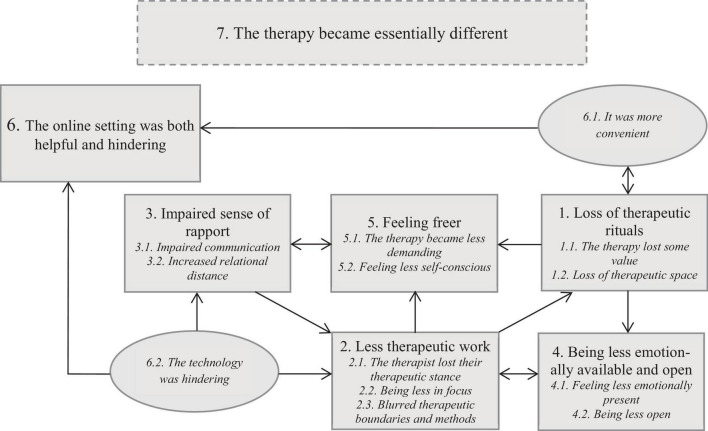
A tentative model of interconnections between themes of transition.

The themes that were identified in the material were connected and influenced each other in different ways (Figure 1). All the interconnected themes (1–6) contributed to and constituted the overarching, elusive experience of telepsychotherapy as something essentially different (7). At the same time, this quality of something different was present in all other themes, thus representing the core and distinctive aspect of the experience of transition.

The online setting was both helpful and hindering (6). It was more convenient (6.1) and practical without all the rituals surrounding the therapy sessions (1), but at the same time therapy lost some value when the intermediate space and time between everyday life and the therapeutic room was absent. Online communication and technological difficulties (6.2) contributed to an impaired sense of rapport (3) and disturbed the therapeutic work (2). This included therapists losing their therapeutic stance, the participants’ feeling of being less in focus, and blurred therapeutic boundaries and methods. On the other hand, being less in focus could create a feeling of being freer (5). The therapy became less demanding and the participant felt less self-conscious. The diminished asymmetry in the relationship could be liberating as it created more relaxed atmosphere. Another aspect that contributed to the experience of less demands was the loss of therapeutic rituals (1). However, frame alternations implied that the therapy felt less valuable and there was less space for emotional presence and for reflection (4). Being less open and emotionally available (4) also made the therapeutic work more difficult and changed the patient-therapist dynamics (2), at the same time as the changed dynamics further reduced the emotional availability. As a result of the impaired sense of rapport (3) it was also more difficult to reach consensus regarding the therapeutic work, which could arose an uncertainty about the goals and means of therapy (2). Blurred therapeutic boundaries and methods made the work less therapeutic, which also reduced the feeling of the therapy as something important ongoing in a sheltered time and space (1).

### Overall View of the Transition

The consensus judgments of the participants’ overall views of the transition to telepsychotherapy, based on the interview material, showed that only one of them presented mainly positive experience, three had mixed, and further three had mainly negative experience. None of the participants expressed a solely positive or negative experience, but the participants assessed as mixed had more explicitly conflicting feelings toward the transition.

### Self-Ratings

The overall results from the participants’ self-ratings are located close to the middle of the 7-point Likert scales and show mixed experiences of the transition to telepsychotherapy. On the group level, the participants were slightly more dissatisfied than satisfied (*M* = 3.86; *SD* = 0.9; range 3–5), they experienced the transition as somewhat more hindering than helpful (*M* = 3.71; *SD* = 1.25; range 3–6), at the same time as they felt rather safe after the transition to the online setting (*M* = 4.29; *SD* = 1.25; range 3–6).

### Personality Orientation and Experiences of the Transition

The self-assessments of personality orientation (PMAI) resulted in six participants categorized as predominantly anaclitic and one participant as predominantly introjective. Due to the skew distribution, no comparisons could be made with regard to personality orientation and the participants’ overall positive-negative view of the transition as well as their self-rated satisfaction, helpful-hindering experiences and feeling safe-unsafe with the transition. However, we can note that the only one introjective participant is represented in most themes and subthemes, inclusive of the overarching, elusive experience of telepsychotherapy as something essentially different, but not in the theme *Feeling freer* (Table 1).

## Discussion

### The Experience of Transitioning to Telepsychotherapy

Previous research could demonstrate that patients generally have a positive attitude toward psychotherapy online (Simpson, 2001; Simpson et al., 2005; Bennett et al., 2020; Watts et al., 2020). However, most of the relevant studies are based on treatments that from start were designed as online psychotherapy. Our study, focusing on the transition from the usual therapeutic frame to telepsychotherapy, partly shows the contrary. On the most general level, the participants’ experiences of transition from the usual therapy setting to telepsychotherapy were marked by the diffuse and elusive feeling that it is something essentially different. This overarching feeling colored all the other interconnected themes that can be seen as embodied and more substantial concretizations of the issue “different how?” The frame alternation and online setting entailed multiple losses for the participants, such as loss of rituals surrounding therapy and of both the sheltered therapy room and the intermediate space and time between the borders of psychotherapy and the everyday life, making telepsychotherapy less cathected and valuable. When several communication channels, accessible with two bodies co-present in the same room, shrank to the screen and/or voice, the contact with the therapist was impaired, the distance in the relationship increased, and the participants felt less emotionally present and open. Less of therapeutic work could be done when the participants felt less in focus for the therapist and especially when the therapist lost their therapeutic stance. The participants’ narratives include sometimes drastic descriptions of the therapist’s boundary crossing, such as “hearing a spray and rubbing… hearing the therapist doing chores at home” and “getting a feeling that she was in several places at the same time.” On the other hand, the participants could feel freer, meeting less demands and being less self-conscious. Attending therapy sessions *via* communication technology could be more convenient but impaired the sense of rapport with the therapist and made both parties more exposed for disturbances.

It is striking that several of the losses experienced by the participants in our study were also reported by therapists in several previous studies (cf. Dores et al., 2020; Rizq, 2020; Essig and Isaacs Russell, 2021; Isaacs Russell, 2021; Weinstein, 2021). In a parallel study (Ahlström et al., 2022), therapists experienced that the loss of the therapy room and of access to non-verbal nuances contributed to impaired contact with the patients and flatter conversations. For some of therapists, at least initially, remote therapy was simply a different therapy (cf. Migone, 2013). Telepsychotherapy could give a therapist unwanted access to their patients’ private space—and give patients access to the therapist’s location when not in their usual office—consequently alternating the patient-therapist dynamic (Isaacs Russell, 2021; Mitchell, 2021). Therapists could admit not being dressed appropriately while seeing their patients out of office (Weinstein, 2021) and report patients abandoning dress code (Sayers, 2021). Furthermore, therapists reported their patients’ difficulties in protecting safe therapeutic boundaries (Isaacs Russell, 2021) and their own insecurity about the patient’s actual presence (Chalker, 2021; Lichtenstein, 2021). In informal discussions and case presentations, therapists often refer to episodes of patients driving car, taking a stroll, or even answering text messages while in session. All the parallel losses experienced by patients and therapists can be seen as corollaries of the impossibility of co-presence of two bodies in the same therapy room and of restricted channels for implicit communication (Brahnam, 2017; Lemma, 2017; Roesler, 2017; Nebbiosi and Federici, 2021). Furthermore, these similarities can be linked to the patients and the therapists sharing the same reality, uncertainties, and fears (Escardó, 2021), the “shared trauma” of pandemic (Nuttman-Shwartz and Shaul, 2021; Tosone, 2021).

In the present study, the participants’ self-ratings showed that they were only marginally dissatisfied with the transition and experienced the transition as slightly hindering, whereas they felt rather safe after the transition. In contrast, thematic analysis revealed several difficulties following the transition. Furthermore, the consensus judgments of the participants’ overall view of the transition, based on the interviews, showed that only one of them had a mostly positive experience. This discrepancy between self-ratings and the interview material might be due to the nature of the data, where the interviews enable a more detailed and nuanced description of subjective experiences, whereas rating scales call for more global assessments expressed in numbers. This might suggest that frame alternations due to the transition did not alter the therapeutic experience as a whole, possibly due to the already established relationship to the therapist acting as a buffer. Nevertheless, the participants expressed their elusive feeling that it is something different with the telepsychotherapy. Likewise, the therapists seem to show a more positive attitude to telepsychotherapy in surveys and rating scales, even when reporting their short-term experiences (Békés and Aafjes-van Doorn, 2020; Dores et al., 2020), than in self-reports and interviews (cf. commentary on 18 papers on therapists’ experiences and reflections from the first year of pandemic by Ellman and Vorus, 2021). The low concordance between qualitative and quantitative evaluations has also attracted attention in outcome studies (Desmet et al., 2021).

With regard to the therapeutic alliance in telepsychotherapy, previous research is not unanimous. A study of client perception of counseling from start designed as online treatment (Leibert et al., 2006) showed that they were generally satisfied and established good working alliance, however, not as satisfied and not as strong alliance as clients in face-to-face counseling. Similarly to our study of transitions to remote therapy, the main disadvantage was the loss of non-verbal cues and personal warmth, whereas the anonymity when disclosing shameful issues was the greatest advantage. In a study of cognitive behavioral therapy for generalized anxiety disorder delivered in videoconference (Watts et al., 2020) patients reported therapeutic alliance as stronger in telepsychotherapy than in conventional psychotherapy. In contrast, Kingsley and Henning (2015) hypothesized that a lack of face-to-face communication may limit the establishment of a strong therapeutic alliance and be a reason as to why telepsychotherapy does not always work. In our material, participants experienced blurred therapeutic frames, as there in many cases were no plain agreements about the goals and means of therapy after the transition. Furthermore, all participants reported increased emotional distance. Deficient agreement about the goals and means of telepsychotherapy, together with the impaired bond with the therapist, is actually impairing the working alliance, as defined by Bordin (1979). Sometimes, the increased relational distance could evoke an experience of the therapist as surreal or dehumanized. On the other hand, some participants perceived themselves as being closer to their therapist when having sessions over the phone. However, this closeness bore a stamp of unclear patient and therapist roles and of blurred boundaries, thus potentially obstructing effective therapeutic work. Still, for some patients the use of communication technology can facilitate being more open and emotionally accessible. As also observed by Chen et al. (2021), patients can feel freer keeping a safe distance in telepsychotherapy, and for some of them remote work can facilitate avoiding dangers of proximity of bodily co-presence. Accordingly, Isaacs Russell (2021, p. 365) described “the online disinhibition effect which leads some patients to become more emotionally forthcoming when treatments are moved onto screens or phones.”

According to Lemma (2017), the online setting in itself is a rupture of alliance; therefore it is of great importance that the therapeutic frames are redefined in accordance with the new situation. The blurred frames in our study might be a result of the frames not being renegotiated and reformulated. One conclusion from our study is the necessity of the therapist’s and the patient’s joint work on the content and meaning of frame alternations due to transitions to telepsychotherapy (and back to the office). Such therapeutic work can be in itself a productive contribution to more effective therapeutic processes.

The participants in our study perceived their therapists as being more distant and less focused on the therapeutic work. They experienced that the therapists could lose their therapeutic stance, the therapeutic boundaries were blurred and the working methods unclear. Accordingly, in a recent study (Probst et al., 2021) patients reported psychodynamic, process-experiential and cognitive interventions as more typical for in-person therapy than for telepsychotherapy, whereas therapists perceived this difference for all examined therapeutic interventions. Dolev-Amit et al. (2021) emphasized that the frame alternation and distance in telepsychotherapy creates new opportunities for enactment and disappointment, thus necessitating development of adjusted supportive techniques for repairing ruptures in the therapeutic alliance. Furthermore, our study demonstrates that the patients experiencing transition to remote therapy pay close attention to the therapists and their behaviors, as well as to subtle changes in the therapeutic collaboration. It is possible that this relational focus is not representative of all patient groups, but the patients focusing on relational factors and boundary crossing might in itself be a consequence of the modified therapeutic setting. Thus, another conclusion from our study is that transitions to remote therapy, for different reasons, make it necessary for the therapists to be especially observant to what happens around borders for the therapy session and to fluctuations in the mutual emotional contact.

Although the self-ratings indicated the participants’ mostly neutral attitude toward the transition to telepsychotherapy, the qualitative analysis clearly revealed that they experienced loss of something essential in therapy, namely the physical presence in the shared therapeutic space and the closeness to the therapist. The positive aspects of the transition seemed to be marginal as the participants consistently expressed their longing back to in-person therapy. The motivation of participants in transitioning to telepsychotherapy was not explored in the present study. However, the general impression from the interviews was that both the patients and their therapists experienced the transitioning as a forced frame alternation. This is also confirmed by the participants’ ratings: they were slightly more dissatisfied than satisfied with the transition and they experienced the transition as somewhat more hindering than helpful, even if they felt rather safe after the transition. For therapists shifting to telepsychotherapy, it can therefore be especially important to reflect on how the sense of emotional presence and attentiveness can be conveyed in telepsychotherapy.

### Personality Orientation and the Transition to Telepsychotherapy

The present study does not allow us to draw any conclusions regarding differences between anaclitic and introjective patients’ experiences, as only one participant was classified as introjective. We can, however, reflect upon the anaclitic patients’ experiences of transition, since the other six participants identified themselves as such. A striking finding is the strong relational focus in the participants’ accounts, which might be understood from the fact that anaclitic patients are more preoccupied with the therapeutic relationship (Blatt and Ford, 1994; Blatt, 2008). A general and prominent theme in our study is the impaired sense of rapport in telepsychotherapy, accompanied by the typical theme of being less in focus and a sense of being uninteresting, replaceable and deprioritized. These worries correspond well to the anaclitic patients’ yearning for warmth, attention, and care in psychotherapy, as well as their fear of abandonment and feelings of loneliness (Blatt and Shahar, 2004b; Levander and Werbart, 2012; Hennissen et al., 2020). Previous research indicated that anaclitic patients, when generally dissatisfied with their therapy, tend to refer to the therapeutic boundaries as an obstacle rather than criticizing their therapist (Levander and Werbart, 2012; Werbart and Levander, 2016). However, the participants in our study attributed shortcomings to their therapists. At the same time, the participants often diminished their critical statements toward the therapist and excused the therapists by referring to the changed circumstances. To sum up, it is possible that our findings are limited to the anaclitic patients’ experiences and new studies are needed to explore the introjective patients’ experiences of the transition.

### Limitations

However, the main limitation is the small sample size, skewed with regard to personality orientation. The recruitment process was more difficult than expected. Even though the recruitment advertisement reached 25,000 Facebook users, only fifteen signed up, of which only seven met the inclusion criteria, and only one of them was classified as introjective. This restricted and skewed self-selection of participants may be due to our narrow inclusion criteria (experiences of transition from in-person to online therapy), but also differences in the anaclitic and the introjective persons’ willingness to talk about their therapy. Relationship oriented anaclitic persons might have a larger need to share their experiences with interested others, whereas introjective persons’ tendency to keep others at a distance might constrain their willingness to participate in a study of their therapeutic experiences (Blatt, 1974; Blatt et al., 2001; Blatt and Shahar, 2004a,b; Blatt and Luyten, 2009; Levander and Werbart, 2012; Hennissen et al., 2020). Moreover, anaclitic persons might have been more dissatisfied with the transition to telepsychotherapy, and their dissatisfaction could be an incentive to share their experiences with a committed interviewer.

A further limitation is the use of prototype matching and self-ratings to classify participants’ personality orientation. The prototype descriptions contain several aspects of one’s personality and it is possible to recognize yourself in some aspects but not others within the same prototype. Furthermore, the concept of personality orientation refers to implicit, deep psychological dimensions that might require clinical expert judgments rather than self-assessments.

### Conclusion and Further Directions

The major strength of this study is the in-depth focus on the patients’ own accounts of their subjective experiences of the transition from in-person to remote psychotherapy, rather than relying on the therapists’ reports about their patients’ reactions. Furthermore, the results are anchored in experiences of patients in both cognitive-behavioral and psychodynamic psychotherapy. The forced transition, due to the COVID-19 pandemic, is an exceptional setting, highlighting the differences between in-person and remote psychotherapy. On the other hand, it is possible that the participants’ experiences of the transition were colored by all changes to the everyday life, caused by the pandemic, thus rendering it more difficult to delimit the phenomenon in focus for this study.

It is our conviction, further supported by our ongoing studies, that the transition strengthened the contrast between in-person and remote psychotherapy, illuminating the relative importance of the therapeutic boundaries and the consequences of frame alternations. For clinicians, learning about the relevance and role of rituals, boundaries and relationship in the therapeutic process, as seen from the patient perspective, will be relevant long time after the pandemic.

A further strength is the strict application of the step-by-step procedure of thematic analysis of qualitative interview data (Braun and Clarke, 2006, 2013) by the two interviewers, blinded with regards to the participants’ personality orientation. The interviewers could approach the material from different points of view and discuss their understanding of the emerging themes. Additionally, the qualitative analysis was continuously audited by the last author.

The data were collected in spring 2021, approximately 1 year after the Public Health Agency of Sweden (Folkhälsomyndigheten [The Public Health Agency of Sweden], 2020) recommended teleworking when possible. Thus our results reflect the patients’ relatively short-term experiences of transition to telepsychotherapy and their ongoing mourning of the loss of the ordinary psychotherapy setting. It is highly likely that both patients and their therapist adjust to the new therapeutic environment and the use of communication technology, both among the patients and among their therapists. It is also probable that the willingness to return to the therapist’s office differ between patients with anaclitic and with introjective personality orientation. Accordingly, the long-term effects of the transition to telepsychotherapy and the different ways back to the customary therapy setting deserve future studies.

Another relevant question for further research is the patients’ (and the therapists’) preferences and experiences of use of audio or video channels in in telepsychotherapy in relation to their personality orientation. Among therapists, perhaps especially among psychoanalytically oriented therapists, telephone seems to be the preferred treatment format for remote psychotherapy (cf. Essig and Isaacs Russell, 2021; Probst et al., 2021). Most patients who experienced transition to telepsychotherapy seem to have explicit preferences for the telephone or video contact, and it might be important for the therapists to follow their patients’ preferences (Ehrlich, 2021; Isaacs Russell, 2021). Different patients might have different needs and the therapists are in need of different competences for video therapy vs. telephone therapy (British Association for Counselling and Psychotherapy, 2021). In our study most participants had experiences of both video and telephone sessions and one of them (anaclitic patient) initiated shift to telephone after initial use of video link. Our sample was too restricted to examine the potential differences in approach.

Further and larger studies, based on both interviews and surveys, are needed for systematic comparisons of anaclitic and introjective patients’ both positive and negative experiences of transition to telepsychotherapy—and back to the therapy room. The patients’ and the therapists’ experiences within the same therapeutic dyads can be related to each other in order to explore the consequences of their convergent or complementary views on the therapeutic process in relation to their convergent or complementary personality orientation (cf. Werbart et al., 2018). However, such a study would demand large number of participants in the different subgroups. An essential topic is also to collect evidence and to test how the impaired sense of rapport when using communication technology can be remedied by adequate, patient-tailored interventions, a topic that has to be included in psychotherapy education and training.

## Data Availability Statement

The raw data supporting the conclusions of this article will be made available by the authors, without undue reservation.

## Ethics Statement

The studies involving human participants were reviewed and approved by the Swedish Ethical Review Authority (registration numbers 2020-06819 and 2021-01188). The patients/participants provided their written informed consent to participate in this study.

## Author Contributions

AW planned and designed the present study, continuously scrutinized the progress of the study, and was responsible for the final version to be submitted. LB and TJ participated in designing data collection and were responsible for recruiting the participants, acquisition of all the data included, primary analysis and interpretation of the data for the work, early drafting, and critical revision in the later stages of the work. BP participated in the research group on transitions to telepsychotherapy and in planning and designing of the present study, continuously scrutinized data analysis, interpretation of results, and early drafting, and contributed with critical revision in the later stages of the work. All authors have given final approval of the version to be published and agreed to be accountable for all aspects of the work.

## Conflict of Interest

The authors declare that the research was conducted in the absence of any commercial or financial relationships that could be construed as a potential conflict of interest.

## Publisher’s Note

All claims expressed in this article are solely those of the authors and do not necessarily represent those of their affiliated organizations, or those of the publisher, the editors and the reviewers. Any product that may be evaluated in this article, or claim that may be made by its manufacturer, is not guaranteed or endorsed by the publisher.
